# Improve screening of HCV infection by targeting high prevalence aged groups: analysis of a cohort of HCV and HIV co-infected patients

**DOI:** 10.7448/IAS.17.4.19601

**Published:** 2014-11-02

**Authors:** Pedro Brogueira, Ana Costa, Ana Miranda, Susana Peres, Teresa Baptista, Isabel Aldir, Isabel Antunes, Fernando Ventura, Fernando Borges, Kamal Mansinho

**Affiliations:** 1 Infectious Diseases, Hospital de Egas Moniz, Lisboa, Portugal; 2 Family Medicine, USF Alpha Mouro, Sintra, Portugal

## Abstract

**Introduction:**

Hepatitis C constitutes a major public health burden. In Portugal, the prevalence is estimated at 1–1.5% [1]. Of these, only 30% are presumed to be diagnosed, which reveals that most infections go unknown. The objective of this study is to identify the age-range distribution at HCV diagnosis and to identify the high-prevalence birth groups that could be targeted for screening, as a strategy to increase diagnosis and identify patients who would benefit most from treatment.

**Methods:**

Retrospective observational study of a cohort of chronic HCV-infected and HIV co-infected patients followed at an Infectious Diseases Center, diagnosed between 1979 and 2014 (Figure 1). Hepatic fibrosis evaluation was performed by real time elastography using METAVIR score. Epidemiological, demographic, clinical, virological and therapeutic data was retrieved from clinical registries. Statistical analysis was performed using Microsoft Excel 2010^®^. Chi2, Student T were used for a significant p value of <0.05.

**Results:**

Our study assessed a cohort of 665 patients: 442 (66.5%) HCV/HIV co-infected and 223 (33.5%) HCV monoinfected. There was a male predominance in both groups (74.9% vs 70.9%). The mean age was 47 HCV/HIV vs 49 years; Portuguese origin in 80% vs 83% and African in 14% vs 12%. The most frequently assumed transmission route was by intravenous drug use (IVDU) (81% vs 72%), followed by sexual contact (18% vs 20%). Mean age at diagnosis was 32 vs 40 years. Mean time since HCV diagnosis was 14, 6 vs 9, 6 years. Fibrosis stage evaluation by real time elastography was available for 133 (30%) and 99 (44.4%) patients (HCV/HIV vs HCV): 16% vs 13% F1; 32% vs 33% F2; 31% vs 35% F3; 21% vs 18% F4. The peak prevalence occurred between the birth intervals of 1960–1969 and 1970–1979 for both groups, corresponding to 81% vs 66,8% (p=0.003) (Figure 1). About three quarters of all patients (76%) were born between the year of 1960 and 1979, with a prevalence of 70% of IVDU.

**Conclusions:**

In our cohort we identify a high risk population for chronically HCV infection, which comprises people born between 1960 and 1979, findings common to those with mono or HIV co-infection. This finding is concordant with the epidemic of IVDU in Portugal around 1980–1990. These patients should be screened for diagnosis in order to be treated and to prevent further disease progression.

**Figure 1:**
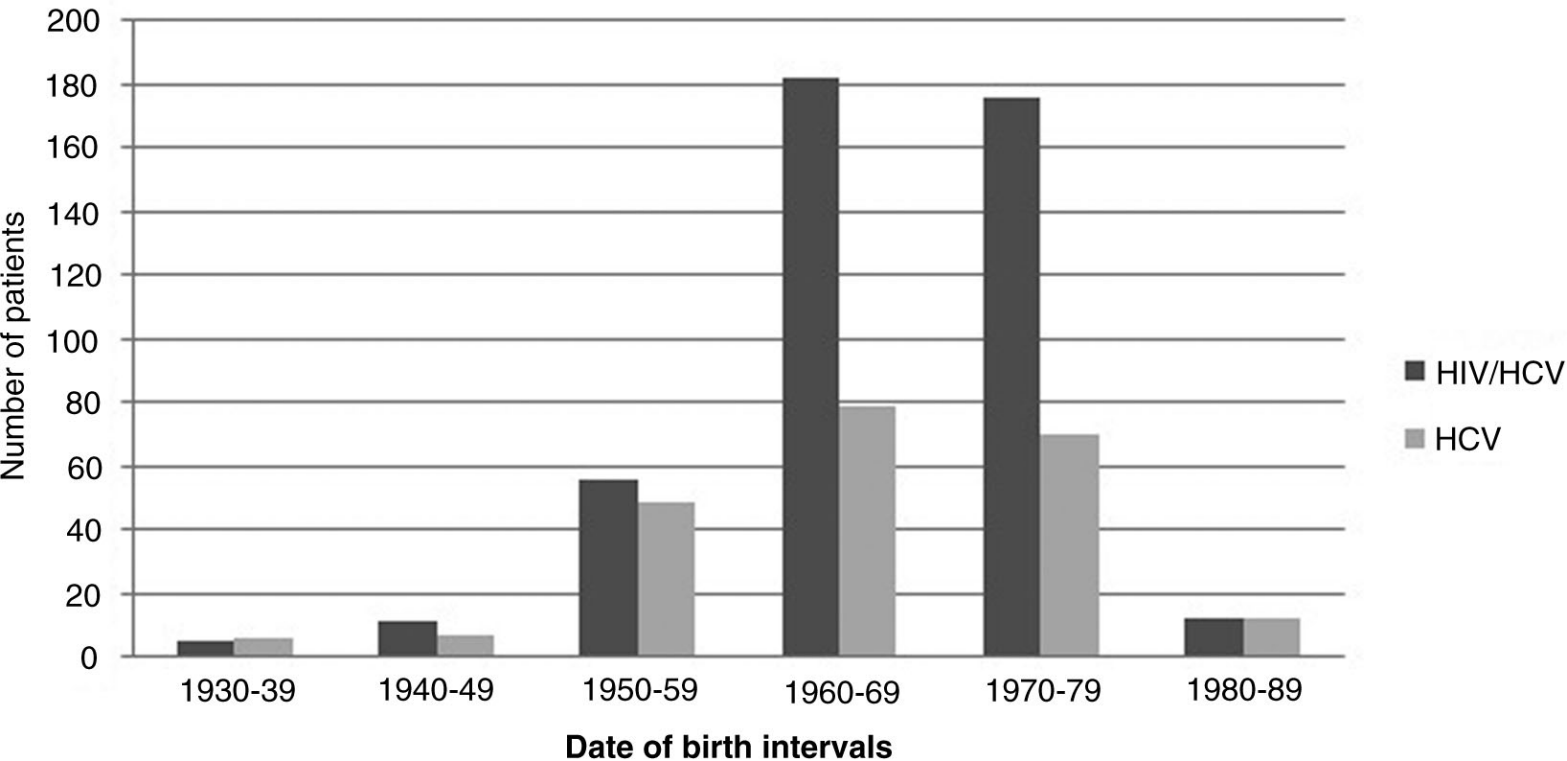
Distribution of HIV/HCV and HCV infected patients by date of birth (n=665).
